# Assessment of Malnutrition, Sarcopenia and Frailty in Patients with Cirrhosis: Which Tools Should We Use in Clinical Practice?

**DOI:** 10.3390/nu12010186

**Published:** 2020-01-09

**Authors:** Benjamin Buchard, Yves Boirie, Lucie Cassagnes, Géraldine Lamblin, A. Coilly, Armando Abergel

**Affiliations:** 1 Service de Médecine Digestive et Hépatobiliaire, CHU Clermont-Ferrand, 63000 Clermont-Ferrand, France; glamblin@chu-clermontferrand.fr (G.L.); aabergel@chu-clermontferrand.fr (A.A.); 2 Service de Nutrition Clinique, CHU Clermont-Ferrand, 63000 Clermont-Ferrand, France; yboirie@chu-clermontferrand.fr; 3 Unité de Nutrition Humaine, UMR 1019 INRA-Université Clermont Auvergne, 63000 Clermont-Ferrand, France; 4 Service de radiologie adultes, CHU Clermont-Ferrand, 63000 Clermont-Ferrand, France; lcassagnes@chu-clermontferrand.fr; 5 Institut Pascal, Thérapies guidées par l’image, UMR 6602 CNRS-SIGMA-Université Clermont Auvergne, 63000 Clermont-Ferrand, France; 6 Centre Hépatobiliaire, AP-HP Hôpital Paul-Brousse, UMR 1193 INSERM-Université Paris Sud, 94800 Villejuif, France; audrey.coilly@pbr.aphp.fr

**Keywords:** malnutrition, sarcopenia, frailty, liver, cirrhosis, liver transplantation, prognosis, body composition, skeletal muscle index, handgrip strength, lean mass, fat mass

## Abstract

Malnutrition is a common comorbidity in patients with cirrhosis. Its prognostic value is indisputable as it greatly affects the evolution of liver diseases. It has a major impact on both morbi-mortality before and after liver transplantation. Being now integrated in the definition of malnutrition and recognized as a new entity in the international classification of diseases, physicians have taken great interest in sarcopenia. Its negative consequences on the fate of patients with cirrhosis are well-demonstrated. The concept of frailty has recently been enlarged to chronic liver diseases as symptoms of impaired global physical functioning. In this article, we will discuss the definitions of malnutrition and emphasize its links with sarcopenia and frailty. We will show the relevance of frailty and sarcopenia in the course of liver diseases. The emerging role of muscle depletion on the cardiorespiratory system will also be highlighted. The importance of body composition will be demonstrated and the main tools reviewed. Finally, we adapted the definition of malnutrition to patients with cirrhosis based on the assessment of sarcopenia together with reduced food intakes.

Malnutrition is frequently found in patients with cirrhosis [1], but its accurate prevalence is still unknown. Indeed, despite the multiplication of tools, the way to evaluate the nutritional status and its severity remains unclear. Basic tools used in the general population such as biomarkers and anthropometric measures are not reliable in chronic liver diseases (CLD), blurred by fluid overload, impaired liver synthetic function and systemic inflammatory state [2,3]. As a result, nutritional assessment is not frequently performed, even in specialized teams. This ascertainment does not question the importance of malnutrition but highlights the need for an international consensus on the definition of malnutrition in CLD.

Interestingly, more than a decade ago, an international expert group has proposed consensus definitions of sarcopenia as a primary alteration in the aging process or as a secondary consequence of chronic diseases [4] so that it has been recently recognized as a new code in the international classification of diseases [5]. Consequently, recent works have increasingly focused on sarcopenia through body composition and highlighted the importance of skeletal muscle depletion in chronic liver diseases [6,7,8,9]. Frailty has also been studied in CLD and considered as symptoms of impaired global physical functioning [10]. However, there is a strong overlap between these three entities. To translate their clinical relevance in practice, borders must be drawn and bridges built. The agreement on tools which should be used in practice will therefore allow designing relevant clinical trials for an optimal nutritional care in this population.

This article will discuss the definitions of malnutrition and emphasize its links with sarcopenia and frailty. It will also show the relevance of frailty and sarcopenia in the course of liver diseases, especially the influence of muscle depletion on the cardiorespiratory system. The importance of body composition will be demonstrated and the main tools reviewed. We finally adapted the definition of malnutrition to patients with cirrhosis based on the assessment of sarcopenia together with reduced food intakes.

## 1. Malnutrition, Sarcopenia and Frailty: Who Is Who?

Malnutrition is defined as a measurable change in physical and mental functions secondary to altered body composition and cell mass, resulting in impaired quality of life and poor clinical outcomes [11]. It is the consequence of insufficient protein and energy supplies. In 2016, the Global Leadership Initiative on Malnutrition (GLIM) endorsed by the European Society for Clinical Nutrition and Metabolism (ESPEN) agreed on a common diagnosis assessment and grading of malnutrition [12]. The diagnosis of malnutrition now requires the combination of phenotypical and etiological criteria. The first criterion consists of reduced food intake and/or assimilation with the presence of acute/chronic disease-related inflammation. The second criterion is based on weight loss, reduced body mass index (BMI) or reduced muscle mass. Malnutrition in patients with cirrhosis is associated with increased complications requiring hospitalization and ultimately leading to death [1,13]. Most of basic tools used to assess nutritional status are not reliable in cirrhosis [2,3]. Liver failure results in decreased rates of albumin, pre-albumin, retinol binding protein and transferrin. Body mass index (BMI) and weight are not reliable measures in case of fluid overload. This ascertainment is important considering that half of patients with cirrhosis waiting for liver transplantation (LT) is registered for decompensated disease [14]. As a consequence, malnutrition in cirrhosis is very difficult to assess. Despite major consequences of malnutrition in patients with CLD, nutritional assessment is not often performed even in hepatology specialized centers. There is then a strong need for dedicated tools, including specific criteria and cut-off points.

Sarcopenia in patients with cirrhosis has been studied for several years. It is defined as a progressive and diffuse loss of skeletal muscle strength, mass and function [11]. Recently, the European Working Group on Sarcopenia in Older People (EWGSOP) proposed a three-step definition of sarcopenia [4]. The diagnosis of sarcopenia is considered as probable in case of decreased muscle strength and confirmed when there is an altered muscle quality and/or quantity. The severity of sarcopenia is lastly evaluated with physical performance (Table 1). The prevalence of sarcopenia was 10% in Child A, 34% in Child B and 54% in Child C in a recent monocentric study [6]. Sarcopenia leads to the development of complications (infections, hepatic encephalopathy, ascites) and poor overall survival [7]. Mortality is high in sarcopenic patients waiting for liver transplantation when compared to non-sarcopenic patients [8]. Many tools have been evaluated in patients with cirrhosis. It is well admitted that malnutrition participates in the onset of sarcopenia but the link between these two nutritional concepts still remains confusing. Sarcopenia is frequently and wrongly considered equivalent to malnutrition, since other causes such as immobilization, endocrine diseases or neurological disorders may also produce sarcopenia. However, it is now integrated in the definition of malnutrition [12] and could therefore fill a need for dedicated nutritional tools in decompensated cirrhosis.

Frailty is a concept widely used in geriatrics and defined as a state of decreased reserve and increased vulnerability relating to physiological decline. It was developed to identify old people at increased risk of poor health outcomes, falls, disability, dependency, hospitalization and mortality [11]. It is based on the evaluation of physical, functional and cognitive capacities. Sarcopenia with malnutrition are important components of frailty. A strong correlation between frailty and malnutrition in old people was recently reported [15]. Ageing is indeed associated with changes in body composition, especially loss of skeletal muscle mass leading to disability and mortality.

Any chronic organ dysfunction can lead to physiological vulnerability. Frailty, especially its physical component, has recently been evaluated in chronic diseases, including chronic liver diseases. Carey et al. early showed that the six-minute walk distance was associated with mortality in liver transplant candidates and inversely correlated with the Model for End-stage Liver Disease score (MELD) [16]. This work opened the path to frailty in liver diseases.

The first question to answer is to know in which order we should evaluate these three parameters. Do we first have to study nutritional status?

## 2. Should We Assess Nutritional State at the Patient’s Bed and How?

**Figure A1:**
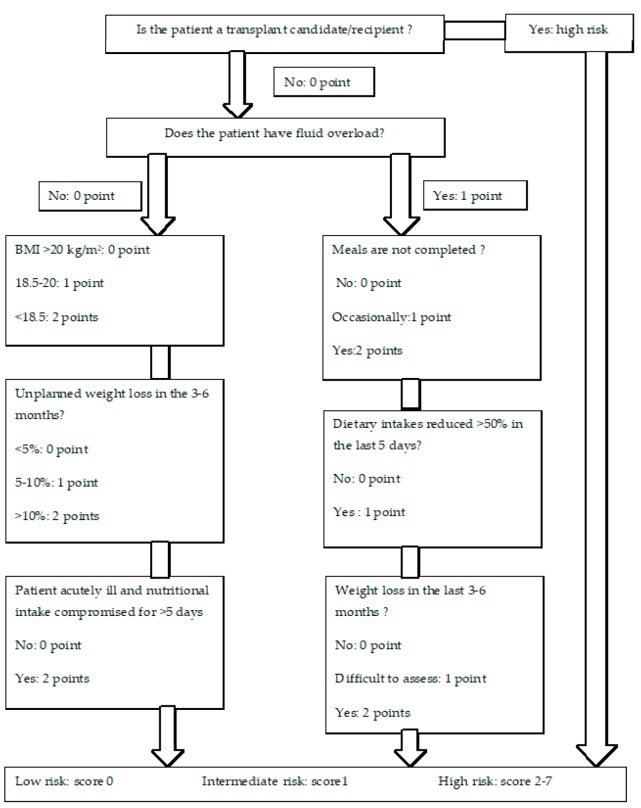
Royal free hospital nutrition prioritizing tool [17]. BMI: body mass index.

The Royal free hospital nutrition prioritizing tool (RFHNPT) is a score developed to screen malnutrition specifically in cirrhosis (Appendix A Figure A1). It was validated compared to the Royal free hospital global assessment (RFHGA), a standard nutritional test in the United Kingdom [17]. The choice of the RFHGA as gold standard is controversial as it combines BMI and arm circumference, which are not reliable in CLD. It was afterwards associated with the occurrence of complications such as ascites, hepato-renal syndrome or hepatic encephalopathy [18]. It was an independent predictor of transplant-free survival and its improvement was associated with better survival. Malnutrition assessed with the RFHNPT also predicted early post-liver transplantation outcomes [19]. More studies worldwide are needed to demonstrate its prognosis value.

The Liver Disease Undernutrition Screening Tool (LDUST) was developed by the American Society for Parenteral and Enteral Nutrition (ASPEN) and the Academy of Nutrition and Dietetics to detect malnutrition in patients with chronic liver diseases evolving for more than three months. Nutritional status was assessed with six questions answered by the patient in less than five minutes, regarding nutrient intakes, weight loss, loss of subcutaneous fat, loss of muscle mass, fluid accumulation and decline in functional capacities (Table A1). It showed a good correlation compared to a dietitian assessment [20,21]. Contrary to RFHPT, LDUST has not been associated yet with clinical issues such as survival and complications appearance. Moreover, LDUST is based on the patient’s statement and lacks objective data.

Current screening tools still have many imperfections and thus cannot be recommended. It has been suggested that patient with Child Pugh C cirrhosis or dry BMI (Appendix A Table A2) <18.5 kg/m^2^ were at the highest risk of malnutrition and should be the ideal target for nutritional assessment and care [22,23]. However, the impact of sarcopenia on survival has been mainly demonstrated for Child Pugh A or B cirrhosis and in patients with low MELD [1,6]. No influence of muscle depletion was found in the most severe patients, probably because their survival is threatened at short term by other factors. The unfavorable prognosis of the most severe cirrhotic patients probably counterbalances the beneficial effects of nutrition [24]. Thus, nutrition care should be started early in the disease course and there is probably an optimal nutritional window to intervene. Hence, we believe that nutritional assessment is mandatory in any patient with cirrhosis, the presence of an evolving chronic liver disease itself being a substantial risk factor for malnutrition. Considering the influence of nutritional status on LT outcomes, it should be specifically evaluated in this population.

As there is no nutritional validated tool, we have to look for other parameters such as body composition and frailty that have been widely studied recently.

## 3. Is Determination of Body Composition in Cirrhosis Helpful to Predict Clinical Outcomes in Patients with CLD?

### 3.1. Which Tools Do Not Work in Chronic Liver Diseases?

Anthropometric measures have been and are still used in daily practice. Mid-arm muscular circumference (MAMC) and triceps skinfold thickness (TSF) are believed to be well correlated with lean muscle mass and body fat. Their predictive value has long been demonstrated; malnourished patients with cirrhosis show lower survival rates [25,26]. However, it can suffer from error measurement due to lack of inter-observer agreement [27]. Fluid overload can affect the accuracy of these anthropometric data. Moreover, recent works, especially in patients with cancer, highlighted their poor correlation with cross-imaging measures [28,29]. Recently, Yao et al. found that MAMC, TSF and BMI were not independently associated with survival in patients with decompensated cirrhosis [30].

Bioelectrical impedance analysis (BIA) is based on the conductivity of hydrated tissues, measure of the volume of total body water and assessment of the lean body mass through an equation. Its use in decompensated cirrhosis is controversial, as Pirlich et al. found a low agreement between body cell mass (BCM) estimated by BIA and BCM derived from total body potassium counting [31]. Phase angle BIA directly measures the integrity of cellular membranes and water cellular distribution without using prediction equation. In a study by Ruiz Margàin et al. [32], it was independently associated with mortality in compensated cirrhosis. Despite its prognosis value, phase angle BIA still needs to be validated compared to gold standard method for the assessment of body composition such as cross sectional imaging. Whole body dual-energy X-ray absorptiometry (DEXA) has also been studied in chronic liver disease. It allows measurement of bone mineral density, fat mass and fat-free mass. Sinclair et al. recently found a large difference in the prevalence of sarcopenia between CT (Computed Tomography) scan (70.3%) and DEXA (38.7%). DEXA underestimates the prevalence of sarcopenia in patients with fluid overload and cannot be used in patients with CLD [33].

### 3.2. How to Correctly Assess Muscle Function and Mass in Patients with Cirrhosis?

As previously explained, ESPEN guidelines [3] now integrate sarcopenia as a major criterion for the diagnosis of malnutrition. The latest EWGSOP guidelines on sarcopenia [4] proposed a new three-step definition of sarcopenia (Table 1). These new guidelines highlight the necessity for both a functional evaluation and quantification of muscle mass.

Firstly, sarcopenia must be suspected in case of loss of muscle strength. Two main tools have been proposed for clinical practice, the hand grip strength (HGS) and the chair stand test (CST). The HGS records the mean value (in kilograms) of three consecutive measurements of the dominant arm gripping a dynamometer. The CST consists in counting the number of times the patient can rise to a full standing position and then sit down in 30 s without any help from the hands. Both tests allow looking for lean mass depletion and low muscle strength. Contrary to the CST, the prognosis value of HGS has been demonstrated in many works in patients with cirrhosis. HGS is an independent factor of mortality [33,34], but weakly correlates with muscle mass and quality assessed on cross-sectional imaging [35]. These results imply that the complementarity of a quantification with a functional approach is needed. As suggested by the authors, new parameters such as muscle function (muscle strength per unit of muscle mass) would be interesting to analyze [35]. Moreover, these tests provide clinical targets to achieve before liver transplantation and even after. An important question remains unanswered about the cut-offs to use. Neither the European Association for the Study of the Liver (EASL) nor the ESPEN in 2018 recommended cut-offs [3,22]. In 2016, the Japan Society of Hepatology published its own guidelines [36] on sarcopenia in liver disease after establishing a working group on sarcopenia assessment criteria. Their choice was based on both their experience and the cut-offs suggested by the Asian Working Group for Sarcopenia (AWGS). A grip strength under 18 kg for women and 26 kg for men were the optimal cut-offs. However, these cutoffs should be taken with caution as the main etiology of cirrhosis in Japan is hepatitis C. In particular, alcoholic liver diseases are at theoretical higher risk of malnutrition due to the direct effects of alcohol and its metabolites on skeletal muscle [37]. The cut-offs could be different but have not been studied in this population.

The second step for diagnosing sarcopenia consists in measuring muscle quantity. CT scan now allows with the help of specific software to quantify the skeletal muscle area (SMA) in cm^2^. SMA is then adjusted to the squared height to obtain the skeletal muscle index (SMI) in cm^2^/m^2^. It has the advantage to be performed easily as the abdomen is the object of diagnostic imaging in cirrhosis. This technique distinguishes ascites from soft tissues. Lumbar skeletal muscle area is relatively well correlated with the whole body muscle mass, especially at the third lumbar vertebra (L3) [38]. It has been previously shown that there was no influence of the software used or of the injection of intravenous contrast [39,40]. In cirrhosis, the SMI has been associated with mortality in patients waiting for liver transplant [6,8]. Optimal cut-offs were set at 50 cm^2^/m^2^ for men and 39 cm^2^/m^2^ for women [8,10]. The choice of these values has been suggested by the EASL [22]. Using the same cut-offs, Banjhi et al. recently found an increased risk of hepatic encephalopathy independently of MELD and also showed that only sarcopenia was predictive of mortality in the multivariate analysis [41]. The cut-offs proposed by the Japan Society of Hepatology [36] are slightly different and were set at 38 cm^2^/m^2^ for women and 42 cm^2^/m^2^ for men. Considering that muscle parameters are likely to be associated with age and ethnicity, it has been suggested to interpret SMI according to reference values in a healthy population [42]. Severe muscle depletion predicts postoperative length of hospitalization but is not associated with survival after transplantation [43]. DiMartini showed that sarcopenia was associated with longer stay, especially in the ICU unit and longer intubation duration [44]. In patients with hepatocellular carcinoma (HCC), low SMI was also associated with mortality and HCC recurrence, independently of cancer stage or Child score [45]. Interestingly, it was demonstrated that muscle mass depletion was more frequent in men and its prognosis value greater than in women [46].

Some authors have suggested to measure the dimensions and surface area of the psoas major. It has the great advantage to be easily feasible because it does not require special software. Golse et al. [47] showed that among many tools including SMI, psoas major area (PMA) at the L3 or L4 level was the best method to predict 1-year post-transplantation mortality. Durand et al. [48] also found significant association between transversal psoas muscle thickness normalized to height and mortality on the liver transplantation waiting list, independently of MELD. However, some physicians pointed out the weaknesses of this tool including poor correlation with the total lumbar muscle area and a high measurement error [49]. Measures of psoas dimensions suppose the use of an anatomic landmark, for instance the umbilicus. The variability of the location of the umbilicus is high between patients and it cannot be used for the measures of single muscle dimensions. The use of an immobile landmark is more appropriate. In a recent work from Ebadi et al., a weak concordance between SMI and psoas muscle index (PMI) was pointed out. PMI corresponds to PMA adjusted to the squared height. Contrary to SMI, no significant association was observed between PMI and mortality in men waiting for liver transplantation [50]. Therefore, a single-muscle method cannot be recommended to assess sarcopenia in chronic liver diseases.

Muscle radiation attenuation (MRA) expressed in Hounsfield Units (HU) is a measure of muscle quality, which is inversely related to muscle fat content and quality. Interestingly, intramuscular fat deposition known as myo-steatosis had a higher prevalence than skeletal muscle loss and was significantly associated with muscle loss [51]. A recent work highlighted the predictive value of myo-steatosis assessed on CT scan for mortality in patients with HCC [52]. In another Japanese cohort, high Intra Muscular Adipose Content (IMAC), low SMI and high visceral to subcutaneous adipose tissue area ratio were independently associated with lower survival [52]. Assessment of muscle quality is therefore a promising tool in liver diseases (Table 2).

Lastly, the severity of sarcopenia can also be appraised with physical performances. Gait speed, short physical performance battery (SPPB), timed-up-and-go test (TUG) or 400 m walk are examples of available tools in geriatrics (Appendix A Table A3). In patients waiting for liver transplantation, physical performances (gait speed and SPPB) have been associated with overall survival [53].

### 3.3. Is Cardio-Respiratory Sarcopenia Frequent in Patients with Cirrhosis and Do They Influence Their Prognosis?

#### 3.3.1. Which Came First: Sarcopenia or Heart Failure?

Cardiac dysfunction in patients with cirrhosis results in poor outcomes and can lead to death [54]. Notably, it is also associated with increased risk of morbidity and mortality following LT [55]. The link between sarcopenia and heart function is of increasing interest. It was demonstrated that patients presenting left ventricular dysfunction following LT had a significant lower muscle mass than those with normal ejection fraction [56]. Some authors hypothesized the bilateral effects of sarcopenia and heart failure (HF) in patients with hypercatabolic diseases [57].

The presence of sarcopenia itself is said to induce heart failure (HF) with the atrophy of the myocardium. This ascertainment was mainly demonstrated in animal models [58]. In patients with cirrhosis, Kazemi-Bajesti showed that sarcopenia was more prevalent in patients with low left ventricular mass [59]. Cardiac sarcopenia was recently defined by the loss of cardiomyocytes resulting in impaired cardiac function in the absence of any cardiovascular disease [60].

Sarcopenia has been mentioned as a frequent comorbidity in patients with HF in the latest guidelines by the European Society of Cardiology [61]. HF induces a significant reduction in physical capacities leading to an increased risk of sarcopenia [62]. It is associated with more severe symptoms and more frequent hospitalization [63]. It is an important predictor of mortality in patients treated for HF [64]. Patients with cirrhosis can be affected with heart failure at different levels. Cirrhotic cardiomyopathy is a common consequence of cirrhosis, affecting about one third of patients [65]. Coronary artery diseases are also frequent comorbidities as cardiovascular risk factors are more prevalent in this population, especially diabetes [66,67].

Relationships between heart failure and depleted lean muscle mass seem indisputable and go both ways. Increased morbi-mortality in sarcopenic patients could be partly explained by this bilateral effect, especially in the post-LT period.

#### 3.3.2. Are Sarcopenic Patients Short of Breath?

As any skeletal muscle, respiratory muscles can be affected by sarcopenia [68]. Sarcopenia is believed to affect patients with cirrhosis by increasing the rate of respiratory infections and the duration of mechanical ventilation in the post-LT period [19,44]. However, works focusing specifically on respiratory muscle strength in sarcopenic patients with cirrhosis are lacking. Most studies were conducted in geriatric populations. Respiratory muscle strength can be assessed with volitional (maximal inspiratory and expiratory mouth pressures) and non-volitional invasive tests (measurements of transdiaphragmatic pressure during phrenic nerve stimulation) [69].

Recently, Ohara et al. showed that sarcopenic patients had lower maximal inspiratory and expiratory pressure. Respiratory muscle strength was inversely associated with parameters of sarcopenia [70]. Sarcopenia was also demonstrated to be associated with peak cough flow in the elderly and hypothesized to have a greater effect on inspiratory muscles [71]. These data could explain the increased rate of respiratory infections through impaired airway clearance and the difficult weaning from mechanical ventilation in sarcopenic patients with cirrhosis but need to be validated in this specific population.

### 3.4. Incorporation of Sarcopenia in MELD: A False Good Idea?

The Model for End-stage Liver Disease is a severity index originally developed to predict three-month mortality following trans-jugular intrahepatic portosystemic shunt (TIPS) placement [72]. It was validated as a strong predictive score of mid-term survival among patients with cirrhosis and adopted for the prioritization of the most severe patients waiting for liver transplantation. Notably, patients with significant portal hypertension are not well served by this score. This is why many derived scores have been proposed, including MELD-sodium, which improved waitlist outcomes in a recent work in the United States [73]. As mentioned above, sarcopenia is associated with mortality in patients waiting for liver transplantation, especially in patients with less severe disease [1,6]. Some models have therefore been developed to accelerate the access to liver transplantation for the most sarcopenic patients. Montano-Loza et al. created a MELD-sarcopenia score with the incorporation of L3 SMI, which predicted mortality better than the original MELD score [74]. The benefit was even greater in patients with low MELD and refractory ascites. Interestingly, the presence of sarcopenia is equivalent to adding 10 points to the MELD score [74]. The predictive value of a MELD-psoas score was also better than that of the original MELD score and equivalent to that of the MELD-Na score [48]. A 15% increase in mortality risk per unit decrease in transversal psoas muscle thickness normalized to height was shown. By combining MELD and handgrip strength, a new model, the MELD-handgrip strength, was the most efficient score to predict waitlist mortality, better than a MELD-CT muscle score [33]. However, cautions must be taken with that kind of scores. We believe that accelerating the access to LT to malnourished patients without a complete rehabilitation should not be a standard of care at the moment. Prospective studies are needed to choose the best moment for transplantation.

### 3.5. Is the Evaluation of Fat Mass Really Useful in Malnutrition Assessment?

Fat mass is composed of subcutaneous adipose tissue (SAT) and visceral fat. Visceral adipose tissue (VAT) is thought to be associated with poor prognosis [75]. Its role in the rising of non-alcoholic steatohepatitis (NASH) prevalence is well-known through insulin-resistance. Being a more vascularized tissue, it contains many immune cells maintaining a state of systematic inflammation and leading to a progressive chronic liver disease [76]. In a study by Fujiwara et al. [45], visceral fat was independently associated with mortality in patients with HCC. In another Japanese study in liver transplantation, high visceral-to-subcutaneous adipose tissue area ratio was predictive of mortality [52]. Ebadi et al. showed that men with cirrhosis had significantly higher VAT but with no impact on mortality [46]. No association was found in women. The predictive role of VAT still needs to be elucidated in cirrhosis as first conclusions go opposite ways.

SAT increase is generally accepted to be more favorable than VAT. Indeed, SAT produces a key hormone, i.e., leptin, which reduces neo-glucogenesis and increases sensitivity to insulin. It was demonstrated that loss of SAT in women was associated with insulin-resistance, dyslipidemia and diabetes [77]. SAT also plays an important role in inflammation by producing adipokines. It is predominant in women, as estrogens favor the growing of fat in subcutaneous areas over visceral tissues [78]. It is therefore not surprising that Ebady et al. [46] found that women had more SAT and that a low SAT index was an independent predictor of mortality. No association was found in men. On the other hand, sarcopenia was not predictive of mortality in women. This work underscores the differences of body composition in men and women and their various effects on survival. The assessment of SAT in women seems to be a promising tool to appraise the relationship between nutrition status and survival in women. More investigations still need to be performed to recommend its use in women.

We showed that the assessment of body composition, especially lean mass, was important for its prognosis value. Nevertheless, physicians also need tools that can evaluate the capacity of the patient to assume daily activities. Is frailty the right tool?

## 4. Are Patients with Cirrhosis Frail?

**Figure A2:**
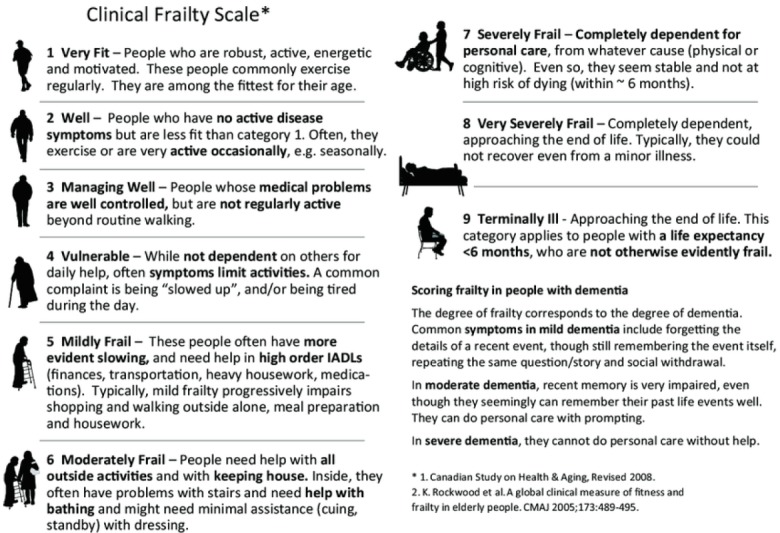
Clinical Frailty Scale adapted from Rockwood et al. [81].

Frailty can be assessed with many tools. It first suffered from a lack of definition. The first score developed in the elderly was the Fried Frailty Index (FFI) [79] (Appendix A Table A4). The frailty phenotype was defined as the presence of three or more criteria: weight loss, self-reported exhaustion, loss of skeletal muscle function, slow walking speed and low physical activity. It was independently predictive of falls, loss of mobility, disability, hospitalization and death [79]. In liver transplantation, its prognosis value was well demonstrated in a study by Lai et al. [53]. It was associated with increased risks of drop-out from the transplantation waiting list and mortality, independently of the severity of cirrhosis. Considering FFI as a time requiring test, the predictive value of the Clinical Frailty Scale (CFS) (Appendix A Figure A2) was then studied [80]. The CFS is divided into nine categories, from 1 (very fit) to 9 (terminally ill), a score over 4 indicating frailty. It has the great advantage to be available, quick and easy to use in daily practice. However, it relies only on the judgement of physicians. In a cohort of 300 patients with cirrhosis [81], frailty defined as CFS > 4 was associated with increased rates of hospitalization and death. The poor correlation between the CFS and the MELD score suggested that frailty evolves independently from the liver disease. In fact, frailty has been found even in compensated disease [82]. Associated with the Montreal Cognitive Assessment (MoCA), the CFS was a very interesting tool to predict the occurrence of hepatic encephalopathy (HE) at 6 months [83]. Lai et al. [84] developed a new tool called the Liver Frailty Index (LFI) to assess frailty by combining grip strength, chair stands (CST) and balance tests (Appendix A Table A5). Using a cut-off set at 4.5, LFI was recently associated with mortality independently of the presence of HE and ascites [85]. Interestingly, not only did physical function worsened in patients waiting for LT, but the baseline physical functions and the longitudinal trajectories of each physical function were significantly associated with mortality [86]. These results [80,81,82,83,84,85,86,87] are crucial as its highlight the weight of frailty in the decision of transplantation and the importance to design studies to improve frailty. We can hypothesize that improving frailty can ameliorate the prognosis of patients with cirrhosis.

A recent consortium from the American society of transplantation proposed a multidimensional assessment of frailty in daily practice [88]. It includes four measures: the Karnofsky index, Activities of Daily Living (ADL), the Liver Frailty Index and the six-minute walk test. This “frailty toolkit” now needs to be validated in an international prospective study.

Can we use frailty as a nutritional screening tool? Can we consider sarcopenia and physical frailty as synonyms? Few works evaluated both sarcopenia and frailty at the same time in liver diseases. In a recent study, Banjhi et al. found that, both in alcoholic and non-alcoholic liver diseases, the prevalence of sarcopenia on CT scans was very different from the prevalence of frailty [89]. Sarcopenia is not identical to frailty but there is a major overlap between definitions and diagnosis criteria of the two phenotypes. Frailty is a more multidimensional concept encompassing not only muscle conditions but also exhaustion, well-being, disability, dependency and cognitive state. Loss of skeletal muscle mass and function remains a strong substratum of frailty, as in malnutrition. The absence of sarcopenia certainly does not rule out frailty but clues of frailty must lead to a complete body composition evaluation. Frailty is a promising concept in nutrition as its multidimensional aspect (physical, mental, social components of frailty) could make physicians consider a more global patient care: nutritional support, deficiencies supplies, physical exercise, screening and treatment of neurological impairment, improvement of the environment and education of caregivers. Seeking minimal hepatic encephalopathy could be relevant in nutritional care of patients with cirrhosis for instance.

## 5. How to Assess Food Intakes?

In patients undergoing nutrition assessment, quantifying intakes is important as intake evaluation is now part of the GLIM definition of malnutrition [12] and influences nutritional care. This evaluation can be easily done by a trained dietitian or physician but is time-consuming. Many technology-based dietary assessment tools exist, relying on self-reported dietary intakes and using either text or images to help identify foods. A majority integrates databases to estimate energy and protein intakes. We are not aware of any work evaluating that type of tools in liver diseases. However, we believe their use could greatly improve nutritional care [90]. A different approach of food intake would be to evaluate the appetite status by using a simple analogic visual scale [91]. Perhaps it would be more appropriate not to consider food intakes on its own, but in regards to individual energy expenditure. Thus, nutritional care would be tailored to fit each situation. Interestingly, a recent meta-analysis highlighted the limited accuracy of predictive equations for resting energy expenditure (REE) in patients with cirrhosis as it mainly underestimates energy needs [92]. The use of indirect calorimetry for an optimal nutritional evaluation was suggested. This attitude seems promising, but more works are needed before making any ascertainment.

## 6. New and Forgotten Tools in Malnutrition

### 6.1. Is Serum Myostatin a Good Reflection of Skeletal Muscle Synthesis?

Myostatin is a growth differentiation factor, member of the *Transforming Growth Factor Beta* protein family, produced and released by myocytes, which inhibit myogenesis by stopping muscle cell growth and differentiation [93]. In a sarcopenic rat model with portocaval anastomosis, increased expression of myostatin was found. Impaired protein synthesis and reduced myocyte function was associated with increased myostatin levels [94]. In a recent study by Nishikawa et al. [95], serum myostatin was independently predictive of mortality in cirrhosis. The authors also found an inverse correlation between serum myostatin and sarcopenia. These data support the future use of serum myostatin to assess muscle depletion in cirrhosis and evaluate response to nutritional interventions.

### 6.2. Branched Chain Aminoacids Normalized to Aromatic Amino Acids Ratio: A Well-Known Criteria but Relevant Way to Appraise Sarcopenia?

Branched chain amino acids (BCAA) are essential amino acids not produced by the human body and brought by diet: leucine, isoleucine and valine. In chronic liver disease, it is well-known that serum concentrations of branched chain amino acids decreased, while the concentrations of aromatic amino acids (AAA) including tyrosine increased, resulting in a decreased ratio of BCAA to AAA, known as the Fischer ratio [96]. This ratio increases as the liver disease progresses and is predictive of survival and other major complications such as hepatic encephalopathy [97]. Decreased values of valine, Fischer ratio and increased rate of phenylalanine and tyrosine were associated with mortality, with a significant correlation with MELD and MELD-Na [98]. The presence of an altered amino-acid ratio was also associated with a reduction in the serum albumin concentration in the following year [99]. Thus, amino acids homeostasis seems to be a relevant nutritional tool and a promising therapeutic target.

### 6.3. Is Muscular Ultrasound Scan Reliable in Cirrhosis?

Cross-sectional areas, muscle thickness (distance between two fasciae), echointensity (homogeneity of muscle) can be measured by ultrasound (US). Tandon et al. showed that right thigh muscle thickness assessed by US scan was the most strongly tool to evaluate muscle depletion and correlated well with gold standard CT scan measures [100]. Ultrasound seems to be a non-invasive, low cost, reliable, reproducible and accurate tool to assess sarcopenia in cirrhotic patients at the bedside. However, many concerns remain regarding the expertise of operators, interpretation in the presence of fluid overload or limitation by poor echogenicity. More work should be done to demonstrate the usefulness of US scan to assess sarcopenia in chronic liver disease.

### 6.4. Magnetic Resonance Imaging: A Non-Radiating Equivalent to CT Scan?

L3 single-slice imaging was found to be the most accurate method to estimate full body skeletal muscular area and adipose tissue on Magnetic Resonance Imaging (MRI) [101]. MRI has the advantage not to carry the risks of radiation. First results from Tandon et al. showed that the intra and inter-observer concordance for the assessment of skeletal muscle obtained from CT versus MRI scans was excellent [102]. Both methods were said to be inter-changeable.

MRI has recently been explored for its ability to distinguish muscle tissue and fat tissue inside the muscle [103]. Fat tissue was subtracted from Muscle Area (MA) measured on the erector spinae to obtain the Fat Free Muscle Area (FFMA). Using gender-specific cut-offs, FFMA was found to be the most accurate model to diagnose sarcopenia compared to CT measures in decompensated cirrhosis. FFMA was independently associated with mortality. Surov et al. [104] recently described higher apparent diffusion coefficient (ADC) values in cirrhosis compared to healthy people. ADC values significantly correlated with the severity of cirrhosis assessed by MELD score. Diffusion imaging can therefore be used to assess muscle quality.

MRI is a promising tool to assess sarcopenia with the use of specific sequences and functional imaging such as diffusion weighted imaging. Recent works proved that it could replace CT scans and improve the ability to detect fat tissue inside the muscle. More investigations are required to generalize its use.

### 6.5. Resonance Magnetic Spectroscopy: An Ancient Non-Invasive Technique to Assess Energy Metabolism with New Perspectives?

Magnetic Resonance Spectroscopy (MRS) is a non-invasive technique based on the excitation of nuclei with radio-waves and the collection of resonance frequencies. Hydrogen is the nucleus of interest most of the time. Different nuclei such as phosphorus, sodium or carbon have been studied. After removing the signal of water, it allows the identification and quantification of metabolites according to their own resonance frequency [105]. However, it has low sensitivity, as only compounds with significant concentration in the body can be detected. This technique can now be associated with MRI in practice.

Phosphorus MRS allows the detection and quantification of many metabolites containing phosphorus, including Adenosine Tri Phosphate (ATP). Corbin et al. early demonstrated that in decompensated cirrhosis, there was a significantly lower level of hepatic ATP compared to compensated cirrhosis and healthy controls, reflecting depleted energy stores in cirrhotic hepatocytes, either due to a lower proportion of functional hepatocytes or increased energy expenditure [106]. Phosphorus MRS was also applied to skeletal muscle [107]. On the anterior tibial muscle of patients with cirrhosis, there was a significant decrease of mitochondrial ATP synthesis, reflected by a decrease in the re-synthesis of phosphocreatine after exercise, in Child Pugh B and C cirrhosis compared to Child A cirrhosis and controls.

MRS, especially focusing on metabolites containing phosphorus, is an interesting noninvasive method to assess energy metabolism both in skeletal muscles and liver of patients with cirrhosis. However, this technique was abandoned in recent research projects. The democratization of MRI in practice must revive our interest in MRS.

## 7. How to Follow Nutritional Status in Patients with Cirrhosis?

Recent guidelines suggested a regular and appropriate follow-up for malnourished and/or sarcopenic patients [22]. However, the way to follow these patients remains unknown. To our knowledge, no dedicated nutritional tool was specifically determined or studied for the follow-up of malnourished patients with CLD. Nevertheless, effects of nutritional interventions have been appraised with various parameters in recent studies.

In a work by Roman et al., patients with cirrhosis practicing physical activity 3 days a week for 12 weeks were followed. Functional capacities significantly increased after exercise. No difference in weight was found between the exercise and the relaxation groups. Anthropometric data did not correlate with changes in muscle mass. These results highlight the poor sensitivity of weight and anthropometric measures to body composition changes [108].

Muscle strength has also been studied in interventional studies. The association of BCAA supplementation and intensive exercise in a small cohort of Japanese patients led to a significant increase in muscle strength [109]. Debette-Gratien et al. observed in a small cohort of 13 patients with cirrhosis following a 12-week adapted physical activity a significant higher knee extensor muscle strength [110]. Testosterone supplementation in patients with hypogonadism significantly increased muscle strength [111]. No study has linked muscle strength to survival after nutritional interventions.

In patients undergoing TIPS, a significant increase in muscle mass on CT scan was found and associated with better survival [112]. Another work showed that cross sectional muscle area of quadriceps measured on MRI significantly increased in a group of patients following resistance training compared to controls [113].

In a cohort of patients with stable liver diseases, a regular practice of physical exercise was associated with increased maximal oxygen consumption (VO2 max) and muscle mass [114]. In their cohort, Roman et al. had found better increase in total effort time and ventilatory anaerobic time in the intervention group [108]. Carey et al. early demonstrated that every 100-m increase in the 6-min walking distance was associated with increased survival [16].

Weight and anthropometric measures are not sensitive enough to detect body composition changes. Sectional imaging is interesting but need further evaluation. Improvement in muscle strength is promising for nutritional follow-up but a link with increased survival must be demonstrated. Functional evaluations like cardiopulmonary exercise test with VO2max assessment or 6-min walking distance are probably the most relevant tools to assess the efficiency of nutritional intervention.

## 8. Conclusions: Malnutrition in Liver Diseases, Towards a More Global and Early Care

Sarcopenia is often detected in the course of chronic liver diseases. It is now integrated in the definition of malnutrition. It fulfills the need for dedicated tools in case of decompensated cirrhosis, when basic tools like BMI and weight loss are not reliable. Based on the GLIM criteria [12], we propose a simple definition of malnutrition in CLD, requiring both sarcopenia along with reduced food intakes (Table 3). The diagnosis of sarcopenia now involves abnormal skeletal muscle function and mass. SMI measured on CT scan and HGS are easily available and objective tools. Their relevance in cirrhosis has been demonstrated in several works and cut-offs have been suggested for practice.

**Figure 1:**
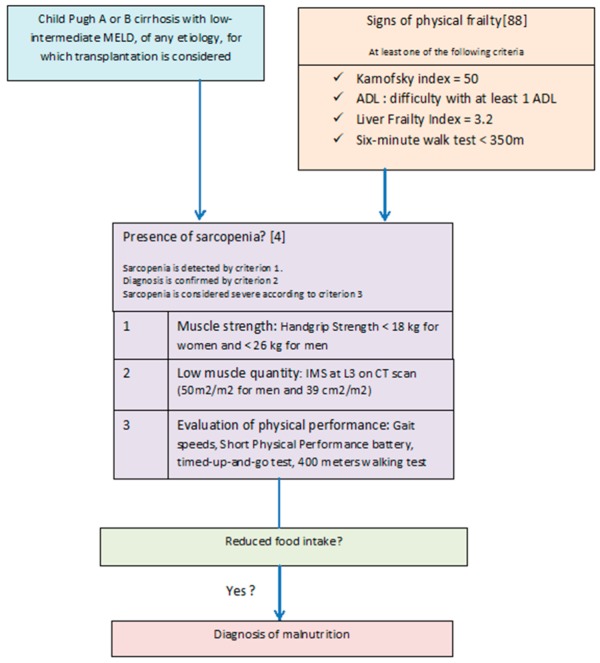
Proposed algorithm for the assessment of malnutrition in cirrhosis.

The prognosis value of sarcopenia is indisputable, especially in the field of transplantation. The role of sarcopenia of the cardiorespiratory system on the morbi-mortality of patients with cirrhosis in general and patients with cirrhosis before and after liver transplantation in particular should be studied more precisely. Frailty has also been deeply studied in liver diseases. It embraces not only muscle conditions and nutritional status, but also cognitive capacities, fatigue, dependency and disability. Thus, the absence of sarcopenia does not rule out frailty but clues of frailty must lead to a body composition evaluation (Figure 1). It is a promising concept in nutrition as its multidimensional aspect makes physicians consider a more global patient care with physical exercise, nutritional support, adaptation to the environment, caregiver’s education and treatment for cognitive impairment such as minimal hepatic encephalopathy.

**Figure 2:**
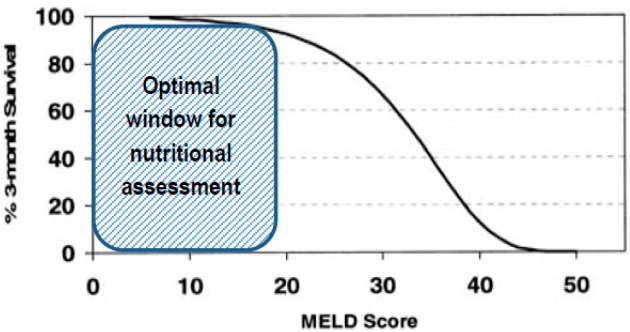
Optimal window for nutritional assessment according to Model for End-Stage Liver Disease (MELD) score and corresponding survival, adapted from Wiesner et al. [115].

Nutritional assessment at the patient’s bed is still far from daily practice considering the imperfections of current screening tools. Sarcopenia seems to have the greatest prognosis value in patients Child A or B with low-intermediate MELD. Therefore, we can question the futility of nutritional assessment in the most severe patients who have poor short term survival. These data unveils the existence of an optimal nutritional window (Figure 2).

## Figures and Tables

**Table 1:** 2018 definition of sarcopenia according to the EWGSOP [4].

Probable sarcopenia is identified by Criterion 1
Diagnosis is confirmed by additional documentation of Criterion 2.
If Criteria 1, 2 and 3 are all met, sarcopenia is considered severe
Criteria 1: low muscle strength
Criteria 2: low muscle quality and/or quantity
Criteria 3: low physical performance

EWGSOP: European working group on sarcopenia in older people.

**Table 2:** Main tools for the assessment of body composition.

Body Mass Component	Tool	Parameter Evaluated	Clinical Relevance
Lean mass	Handgrip strength (kg): mean value of three consecutive measurements of the dominant arm gripping a dynamometer	Muscle function	Predictive of mortality in the LT waiting list [33,34] Decrease of the HGS value with the severity of cirrhosis [34]
	Skeletal muscle index on CT scan (cm^2^/m^2^): semi-automatic measure of skeletal muscles at L3 using HU thresholds of −29 to +150 normalized to the square of height	Muscle quantity	Predictive of mortality in the LT waiting list, especially in men [6,8] and in patients with HCC [45] Predictive of post-LT complications: longer ICU stay, longer hospital stay, higher days of intubation, increased risk of infections [19,44]
	Myosteatosis on CT scan (HU): attenuation of skeletal muscle radiation at L3	Muscle quality	Significant association with skeletal muscle depletion [51] Predictive of mortality in the LT waiting list [52]
Fat mass	Visceral adipose tissue on CT scan (cm^2^): semi-automatic measure at L3 using HU thresholds of −150 to −50	Visceral fat	Association with mortality in patients with HCC [45]
	Subcutaneous adipose tissue on CT scan (cm^2^): semi-automatic measure at L3 using HU thresholds of −190 to −30	Subcutaneous fat	Predictive of mortality in women waiting for LT [46]

LT: liver transplantation, HGS: handgrip strength, CT: computed tomography; ICU: intensive care unit, HU: Hounsfield unit, HCC: hepatocarcinoma.

**Table 3:** 2018 definition and grading of malnutrition adapted for chronic liver disease [12].

Phenotypic Criteria	Etiologic Criteria	
Reduced skeletal muscle index on CT scan at L3 level 50 cm^2^/m^2^ for men 39 cm^2^/m^2^ for women 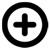 AND Altered muscle strength Handgrip strength < 18 kg for women and < 26 kg for men	Reduced food intake assessed by expert dietician or physician 50% of energy requirements > 1 week OR Any reduction for >2 weeks *	Evolving chronic Liver Disease

* Total energy intake of 35 kcal/kg/day in cirrhosis for nutritional improvement. CT: computed tomography.

## Abbreviations

CLD	Chronic Liver Diseases
GLIM	Global Leadership Initiative on Malnutrition
ESPEN	European Society for Clinical *Nutrition* and Metabolism
BMI	Body Mass Index
LT	Liver Transplantation
EWGSOP	European working group on sarcopenia in older people
MELD	Model for End-stage Liver Disease
RFHNPT	Royal Free Hospital Nutrition Prioritizing Tool
RFHGA	Royal Free Hospital Global Assessment
LDUST	Liver Disease Undernutrition Screening Test
ASPEN	American Society for Parenteral and Enteral Nutrition
MUST	Malnutrition Universal Screening Tool
MAMC	Mid-arm muscular circumference
TSF	Triceps skinfold thickness
BIA	Bioelectrical Impedance Analyses
BCM	Body Cell Mass
CT	Computed Tomography
DEXA	Dual-Energy X-ray Absorptiometry
HGS	Hand Grip Strength
CST	Chair Stand Test
EASL	European Association for the Study of Liver
AWGS	Asian Working Group for Sarcopenia
SMA	Skeletal Muscle Area
SMI	Skeletal Muscle Index
L3	third lumbar vertebra
HCC	Hepatocellular Carcinoma
PMI	Psoas Muscle Index
MRA	Muscle Radiation Attenuation
HU	Hounsfield Units
IMAC	Intra Muscular Adipose Content
SPPB	Short Physical Performance Battery
TUG	timed-up-and-go test
HF	Heart Failure
TIPS	Transjugular Intrahepatic Portosystemic Shunt
SAT	Subcutaneous Adipose Tissue
VAT	Visceral Adipose Tissue
NASH	Non-Alcoholic Steatohepatitis
HOMA-IR	Homeostasis Model Assessment of Insulin Resistance
FFI	Fried Frailty Index
CFS	Clinical Frailty Scale
MoCA	Montreal Cognitive Assessment
LFI	Liver Frailty Index
ADL	Activities of Daily Living
REE	Resting Energy Expenditure
BCAA	Branched Chain Amino Acids
AAA	Aromatic Amino Acids
BTR	BCAA to Tyrosine Ratio
MRI	Magnetic Resonance Imaging
ADC	Apparent Diffusion Coefficient
FFMA	Fat Free Muscle Area

## Appendix A

**Table A1:** Liver disease undernutrition screening tool [20].

Patient Questions	Column A	Column B	Column C
How have you been easting lately?	Normal or fine I’ve been trying to eat less than normal	I’ve been eating less than normal for a month or less I don’t know	I’ve been eating less than normal for more than one month
Have you lost any weight in the last year?	No Yes, but I’ve been trying to lose weight	Yes, I’ve lost some weight I don’t know	I’ve lost a lot of weight
Have you noticed any loss of body fat or thinning or your arms or ribs?	No	Yes, a little I don’t know	Yes, a lot
Have you noticed any muscle loss in you temples, legs, clavicles or shoulders?	No	Yes, a little I don’t know	Yes, a lot
Do you have any fluid or swelling in your abdomen or legs?	No	Yes, I have some fluid I don’t know	Yes, I have a lot of fluid
Are you able to participate in your usual activities?	Yes, I can participate in all my usual activities	No, occasionally I am too tired, weak, or feel too bad I don’t know	No, often I am too tired, weak, or feel so bad that I cannot participate

5 or more boxes checked in column A, No undernutrition; 2 or more boxes checked in column B or C, Undernutrition identified.

**Table A2:** Calculation of dry weight [116].

	Ascites	Œdema
Minimal	2.2 kg	1 kg
Moderate	6 kg	5 kg
Severe	14 kg	10 kg
Dry weight (kg) = real weight adjusting for ascites and peripheral *œ*dema		

**Table A3:** Evaluation of physical performances.

Gait speed [117]	Measure of walking speed (meter/second)
Short performance battery [118]	Balance test (seconds) Chair stand test (seconds) Walking speed on a four-meter distance (meter/second)
Timed-up-and-go test [119]	Time taken by an individual to stand up from an armchair, walk a distance of three meters, turn, walk back to the chair, sit down (seconds)
400-m walk test [120]	Time and ability to complete a 400-m walk (seconds)

**Table A4:** Fried frailty index derived from Cardiovascular Health Study [79].

Criterion	Frailty Status
Shrinking	Frailty cut point: Baseline: Self-reported unintentional weight loss ≥10% in previous year Follow-up: Unintentional weight loss ≥5% of previous year’s body weight or BMI < 18.5kg/m^2^
Physical endurance/energy	Geriatric Depression Scale: 1. Do you feel full of energy? 2. During the last 4 weeks how often you rested in bed during day? Response options: Every day, every week, once, not at all. Frailty cut point: No to 1 and every day or every week to 2.
Low physical activity	Frequency of mildly energetic, moderately energetic and very energetic physical activity. Response options: ≥3 times per week, 1–2 times per week, 1–3 times per month, hardly ever/never Frailty cut point: Hardly ever/never for very energetic physical activity and for moderately energetic physical activity.
Weakness	Hand grip strength (kg): dominant hand, average of 3 measures. Frailty cut point: Grip strength: lowest 20% (by gender, body mass index) Men BMI ≤24 ≤29 BMI 24.1–26 ≤30 BMI 26.1–28 ≤30 BMI >28 ≤32 Women BMI ≤23 ≤17 BMI 23.1–26 ≤17.3 BMI 26.1–29 ≤18 BMI >29 ≤21
Slow walking speed	Walking time in seconds over 15 feet Frailty cut point: Slowest 20%, stratified by gender and median standing height. Men Height ≤173 cm ≥7 s Height >173 cm ≥6 s Women Height ≤159 cm ≥7 s Height >159 cm ≥6 s OR Time to complete “timed up and go test” (TUG) Frailty cut point: TUG time ≥19 s

Frail: ≥3 criteria present; Intermediate or Pre-Frail:1 or 2 criteria present; **Robust:** 0 criteria present.

**Table A5:** Liver frailty index [84].

Clinical Parameters	Coefficients
Sex-adjusted grip strength (three attempts with the dominant arm)	Multiplied by −0.33
Chair stand test (seconds to do 5 chair stands)	Multiplied by −2.529
Balance test (seconds holding three positions)	Multiplied by −0.04
	+6
Total score	Frailty if ≥4.5

See also https://liverfrailtyindex.ucsf.edu/.
